# A bromoform based Investigational Veterinary Product fed twice daily to lactating dairy cows had no adverse effects on eating, rumination, or locomotion behaviours

**DOI:** 10.1016/j.vas.2025.100539

**Published:** 2025-11-10

**Authors:** R. Tognelli, P.S. Alvarez-Hess, A.S. ó Neachtain, S. Chandra, S.R.O. Williams, S. Jacques, S.E. Denman, R.J. Eckard, J.L. Jacobs

**Affiliations:** a Agriculture Victoria Research, Department of Energy, Environment and Climate Action Ellinbank, Victoria, 3821, Australia; b School of Agriculture, Food and Ecosystem Sciences, Faculty of Science, The University of Melbourne, Victoria, 3010, Australia; c Agriculture Victoria Research, Department of Energy, Environment and Climate Action, Tatura, Victoria, 3616, Australia; d Rumin8 Pty Ltd, Suite 1, Level 2, 66 Kings Park Road, West Perth 6005, WA, Australia; e CSIRO, Agriculture Flagship, Queensland Bioscience Precinct, St. Lucia, QLD, Australia; f School of Applied Systems Biology, La Trobe University, Bundoora, Victoria, 3086, Australia

**Keywords:** Animal welfare, Ruminants, Feed additives, Methane mitigation

## Abstract

An Investigational Veterinary Product (IVP) containing synthetic bromoform has emerged as a promising technology for reducing livestock enteric methane emissions. However, the effects of feeding the bromoform-based IVP on eating, rumination and locomotion behaviour (critical animal welfare indicators) remain unclear. This study assessed the response of supplementing twice daily an IVP at two bromoform inclusion rates to lactating dairy cows at milking on i) eating and rumination behaviour; and ii) locomotion behaviour. Thirty multiparous, Holstein-Friesian cows (214 ± 15.2 days in milk) were allocated to one of three treatments; CON (0 mg bromoform/cow per day), LowBR (227 mg bromoform/cow per day) and HighBR (455 mg bromoform/cow per day). The IVP was mixed with 3.1 kg dry matter of grain and offered during each milking. The basal diet consisted of vetch hay offered *ad libitum*. Cows were equipped with a RumiWatch System noseband pressure sensor and pedometer (ITIN + HOCH GmbH, Liestal, Switzerland) for 11 days. Data were analysed by CRD-based repeated measures split-plot ANOVA using Genstat 24 (VSN International, Hemel Hempstead, UK). Results showed no significant effect of the IVP on eating, rumination, lying or standing behaviour. Although cows fed the IVP showed an increase in walking duration, the difference was negligible in practical terms (twelve minutes per day). These findings indicate that feeding dairy cows with the bromoform-based IVP does not change their behaviour, resulting in a potential technology to reduce enteric methane emissions without compromising animal welfare.

## Highlights

• A bromoform-based additive did not affect dry matter intake or eating behaviour.
• Daily eating, ruminating, and activity patterns were unaffected by the diets.
• Lying, standing, and walking were not affected by the treatment diets.
• A bromoform-based additive had no negative effect on dairy cow welfare.

## 1 Introduction

Over the past decade, efforts to reduce the environmental footprint of ruminants have increasingly focused on feeding strategies to mitigate enteric methane emissions. Methane inhibitors such as 3-nitrooxypropanol (3-NOP) and *Asparagopsis* spp*.*, which inhibit crucial enzymes in methanogenesis, have demonstrated significant effectiveness (Kebreab et al., 2023, 2025). *Asparagopsis* based additives have reduced methane yield by up to 44 % in dairy cows (Alvarez-Hess et al., 2023) and up to 98 % in beef cattle (Kinley et al., 2020). Similarly, 3-NOP has been reported to reduce methane yield by an average of over 30 % in dairy cows (Kebreab et al., 2023) and up to 87 % in beef cattle (Almeida et al., 2023). Despite their environmental benefits, there is limited information on the effect of these methane inhibitors on rumen function, animal behaviour, dry matter intake (DMI), health, or overall production (Llonch et al., 2017).

Changes in ingestive and locomotion behaviours are key indicators of dairy cow welfare and productivity. These behavioural changes usually happen in response to social or environmental changes (Huzzey et al., 2006; Costa et al., 2016; Crossley et al., 2017), or variations in feed characteristics and availability (Maekawa et al., 2002; Beauchemin & Yang, 2005; Grant & Ferraretto, 2018), affecting DMI and time spent eating and ruminating. Monitoring ingestive behaviours can provide valuable information relating to dairy cows' health disorders, such as subclinical metritis (Urton et al., 2005; Huzzey et al., 2007), mastitis (Antanaitis et al., 2022), ketosis (González et al., 2008), and subclinical acidosis (DeVries et al., 2009; Antanaitis et al., 2024). Similarly, changes in locomotion behaviour, including walking, standing and lying duration, are widely used as welfare indicators (Müller & Schrader, 2003) and are associated with cow comfort (Haley et al., 2000) and milk production (Elischer et al., 2013). Despite its importance, there is limited research on how methane inhibitors may affect these behavioural parameters.

The few studies that have included measures of animal behaviour when fed 3-NOP (Vyas et al., 2018; Kim et al., 2019; Maigaard et al., 2024) or *A. taxiformis* (Nyløy et al., 2023) have shown contrasting results. Altered feeding behaviour and reduction in DMI were reported when 3-NOP (Maigaard et al., 2024) and *A. taxiformis* (Nyløy et al., 2023) were fed to dairy cows. Conversely, other studies reported no significant effects of 3-NOP supplementation on feeding behaviour (Vyas et al., 2018; Kim et al., 2019) or locomotion behaviour (Kim et al., 2019) in beef steers.

Recently, an Investigational Veterinary Product (IVP) containing stabilised synthetic bromoform has shown methane mitigation of 95 % with no negative effects on DMI (Kelly et al., 2025). While these findings suggest that the IVP does not compromise rumen function, limited research exists on its effects beyond intake and production outcomes. In particular, no studies have examined ingestive or locomotion behaviour in dairy cows fed synthetic bromoform-containing additives. These measures provide more sensitive indicators of animal welfare and can capture subtle responses that DMI alone may overlook. Such a gap in knowledge makes it difficult to fully assess any potential animal welfare implications of feeding these additives. This knowledge is increasingly important as society expects animal welfare to be an integral part of sustainable livestock production (Broom, 2010). A growing number of consumers associate animal welfare with higher product integrity and quality (Cornish et al., 2019) and consider it a key factor when purchasing food products sourced from animals (Clonan et al., 2015; Cornish et al., 2019). Therefore, it is essential to identify and quantify the impact of anti-methanogenic feed additives on animal health and welfare. This approach will facilitate a well-informed discussion on selecting strategies to improve the environmental sustainability of livestock production while ensuring animal welfare is not compromised.

The objective of this experiment was to evaluate the response of feeding an IVP containing two rates of bromoform to lactating dairy cows as a supplemental feed offered twice daily at milking on i) eating and rumination behaviour; and ii) locomotion behaviour. We hypothesised that i) offering either rate of bromoform in the IVP twice daily at milking would have no effect on eating and rumination behaviour, and ii) regardless of the rate of bromoform IVP fed to dairy cows, there would be no difference in locomotion behaviour.

## 2 Materials and methods

The study was carried out at the Agriculture Victoria Research, Ellinbank SmartFarm, Victoria, Australia (38°14′S, 145°56′E).

### 2.1 Experiment design, cows and treatments

Thirty multiparous, Holstein-Friesian cows with an average milk yield (MY) of 23.7 ± 3.05 kg milk/day (mean ± standard deviation), at 3.7 ± 1.75 parities, 214 ± 15.2 days in milk (DIM), and a liveweight of 626 ± 53.2 kg were used in the experiment. The experiment had three treatment diets; 1) CON: basal diet, 2) LowBR: basal diet plus 106 mL/day of low-bromoform based IVP (bromoform concentration 2.14 mg/mL), 3) HighBR: basal diet plus 106 mL/day of high-bromoform based IVP (bromoform concentration 4.29 mg/mL). The IVP was supplied by Rumin8 Pty Ltd (West Perth, WA, Australia) and developed using their patented procedure. The basal diet consisted of *ad libitum* vetch (*Vicia sativa* L.) hay, offered using electronic feed bin system, and 6.2 kg dry matter (DM)/day of a grain mix offered twice daily in the milking parlour. The grain mix consisted of rolled barley grain (296 g/kg DM), cracked corn grain (280 g/kg DM), solvent extracted canola meal (206 g/kg DM), rolled wheat grain (148 g/kg DM), molasses (15.0 g/kg DM), sodium bicarbonate (15.0 g/kg DM), limestone (10.0 g/kg DM), and minerals (28.6 g/kg DM).

Cows in the CON treatment were offered 0 mg bromoform per day, cows in the LowBR group were offered 227 mg bromoform per day and cows in the HighBR group were offered 455 mg bromoform per day. The doses were selected based on previous studies that have fed *Asparagopsis* to dairy cows and showed methane mitigation responses (Roque et al., 2019, 303 and 420 mg bromoform/cow per day; Alvarez-Hess et al., 2023, 480 mg bromoform/cow per day; Alvarez-Hess et al., 2024, 266, 398 and 432 mg bromoform/cow per day).

A completely randomised design (CRD) was used in the experiment, with 10 cows allocated per treatment, using the COVDESIGN procedure in GENSTAT 24 (VSN International, Hemel Hempstead, UK). Groups were balanced for MY (considering the mean of the 7 days prior to the experiment), liveweight (mean of 14 measures recorded prior to the experiment using walkover weigh scales (AWS100 walkover scales, DeLaval International, Tumba, Sweden), DIM at the beginning of the experiment, and parity.

### 2.2 Feed allocation and intake measurement

All cows were offered a common diet during the pre-experimental period.

From experiment days 1 to 3, cows had access to perennial ryegrass (*Lolium perenne* L*.*) pasture (∼20 kg DM/day of available feed) and were fed 6.2 kg DM per day of the grain mix as already described.

From experiment days 4 to 18, cows were fed *ad libitum* vetch hay and gradually transitioned onto the target IVP rates in four 3-day steps, 25, 50, 75, and 100 % of the full dose.

From experiment days 18 to 28, eating, rumination and locomotion behaviour were measured using the RumiWatch System (ITIN + HOCH GmbH, Liestal, Switzerland).

Throughout the experiment, individual cow hay intakes were measured using feed bins mounted on load cells, which were electronically linked to the cows’ identification tags (Gallagher Animal Management Systems, Hamilton, New Zealand). To prevent rain from affecting measurements, the feed bins were placed under a small roof. All feed bins were calibrated before the start of the experiment. Between milking events, cows had unrestricted access to the feed bins and loafing area, with water freely available from a water trough located in the loafing area.

The bromoform-based IVP was mixed with each cow’s individual grain ration once daily in the afternoon, then sealed in plastic bags for distribution at that day’s afternoon milking and the following morning milking. 3.1 kg DM of grain mix was delivered in the milking parlour at each milking. Cows were allowed 20 min to consume their ration; any refusals were collected and weighed, with the proportions of components assumed to match those of the offered grain mix. Representative samples from both the vetch hay and grain mix were collected to determine DM concentration and calculate DMI.

Dry matter concentration was determined by drying feed samples in a forced draft oven at 105 °C for 48 h (AOAC International, 2000; method 930.15). Samples of each feed were collected for compositional analysis, stored at −18 °C, bulked within feed type over the measurement period, oven-dried at 60 °C for 72 h, and ground to pass through a 0.5 mm screen. Feeds were analysed for concentrations of crude protein (AOAC International 2000; method 990.03), acid detergent fibre (AOAC International 2000; method 7.074), neutral detergent fibre (AOAC International 2000; method 2002.04), lignin (AOAC International 2000; method 949.04), non-fibre carbohydrates (AOAC International 2000; method 992.09), starch (AOAC International 2000; method 996.11), ash (AOAC International 2000; method 942.05), crude fat (AOAC International 2000; method 2003.05). Concentrations of estimated metabolisable energy (ME) were calculated using Eq. (1) (NRC, 2001) (Table 1).

(1) ME(MJ/kgDM)=((1.01×DE)−1.88+0.019x(EE−3))

Where DE is digestible energy in MJ/kg DM and EE is ether extract as a percentage of DM.

**Table 1:** Nutrient composition of main dietary ingredients offered (g/kg DM unless otherwise stated).

Item	Grain mix	Vetch hay
Crude protein	174	203
Acid detergent fibre	84	370
Neutral detergent fibre	141	433
Lignin	28	77
Non-fibre carbohydrates	575	224
Starch	433	20
Ash	78	109
Crude Fat	32	31
Metabolisable energy (MJ/kgDM)	12.9	11.3

### 2.3 Eating and rumination behaviour

Eating and rumination behaviour were recorded for 11 days (experiment days 18 to 28). During the recording period, all cows were fitted with RumiWatch noseband sensors from the RumiWatch System (ITIN + HOCH GmbH, Liestal, Switzerland). The operational procedures and specifications of the RumiWatch System have been outlined in previous studies (Zehner et al., 2012, 2017). Briefly, the noseband sensor consisted of a silicone pressure tube filled with glycol and an integrated pressure sensor positioned within a polyethylene halter across the cow’s nasal bridge. The pressure sensor was linked to a data logger, which was secured in a protective plastic case on the right side of the halter. Two 3.6-V batteries, supplying power, were attached to the left side of the halter. The data logger recorded both the pressure variations detected by the sensor and the triaxial acceleration of the halter at a frequency of 10 Hz.

At the end of the recording period, the data were downloaded, processed with the RumiWatch Manager (version 2.1.0.0, Itin+Hoch GmbH, Liestal, Switzerland) and converted to a CSV format using the RumiWatch Converter software 0.7.4.13. The RumiWatch Converter software utilised algorithms to convert the recorded pressure data into classified measurement data of animal activity, specifically eating, ruminating drinking, and other activity times. This procedure contained raw classification summaries in 1-min resolution, which were then aggregated into 1-hour periods.

### 2.4 Locomotion behaviour

During the recording period, cows were equipped with a pedometer (RumiWatch System, ITIN—HOCH GmbH, Liestal, Switzerland) to assess locomotion behaviour. Following the manufacturer's instructions, the pedometers were attached to the cow’s left hind leg, positioned above the metatarsophalangeal joint. The RumiWatch pedometer is a system based on accelerometers designed to measure cows' lying, standing, and walking times (Kajava et al., 2014). At the end of the experimental period, raw data were downloaded and converted to a CSV format using RumiWatch Converter software 0.7.4.13. Data were aggregated into periods of 1 h.

### 2.5 Calculations and data summary

Data gathered during the recording period were summarised in daily time budgets (h/d) for each individual behaviour. Additionally, to demonstrate the impact of the IVP and feed delivery times on the circadian patterns of behavioural activities, the time spent eating, rumination, and other activities was presented using data aggregated into 1 to 24-h intervals (min/h).

Two cows from the HighBR treatment were removed due to illnesses unrelated to the experiment. Four RumiWatch noseband sensors and three pedometers failed to record due to battery depletion and required replacement. Therefore, complete noseband sensor data were obtained from 24 cows over 11 days, while pedometer data were successfully collected from 25 cows over the same period.

Eating index (h/kg DMI) was calculated by dividing the total eating time (h/d) by the total DMI (kg DM/d). Total DMI was calculated by summing the amount of vetch hay consumed (as measured in the feed bins) and the amount of grain fed per day, taking into consideration any refusals in the milking parlour. Rumination index (h/kg DMI) was calculated by dividing the total rumination time (h/d) by the total DMI (kg DM/d) (Nyløy et al., 2023).

Chewing index (h/kg DMI) was calculated by dividing the total eating and rumination time (h/d) by the daily DMI (kg DM/d). The calculation for the number of chews per unit DMI involved dividing the total eating and ruminating chews (counts/d) by the daily DMI.

### 2.6 Statistical analysis

Following Maceina et al. (1994), data were subjected to CRD-based repeated measures split-plot analysis of variance (ANOVA) based on linear model (2), accounting for the effects of the four pre-treatment measured covariates (Cov 1= CoV1Milk_7day, Cov 2= CoV2BW_7day, Cov 3= CoV3DIM, Cov 4= CoV4Parity) and the temporal correlation that may occur among repeated measurements made on cows at different days.

(2) Yijk=μ+Tj+Cov1+Cov2+Cov3+Cov4+εij+Pk+(TP)jk+εijk

Where Yijk is the data corresponding to the i th cow and j-th treatment (*j* = 1, …, 3) at k-th day (*k* = 1, …, 11), µ is general mean, Tj is the effect of j-th treatment, εij is the between-cows residual term, Pk is the effect of k-th day, (TP)jk is the effect of the interaction between j-th treatment and k-th day, and εijk the within-cows residual term. The two residual terms εij and εijk were assumed to be normally distributed with zero mean and constant variance as required for a valid application of ANOVA, which was found to hold good as indicated by the ANOVA diagnostic plots of residuals after fitting model. The between-cows residual term εij forms the basis for testing the statistical significance of the effect of treatment. The within-cows residual term εijk forms the basis for testing the statistical significance of the effects of period and its interactions with treatment.

Data were analysed with Genstat 24th edition (VSN International Ltd., England, UK).

## 3 Results

### 3.1 Eating and rumination behaviour

**Fig. 1:**
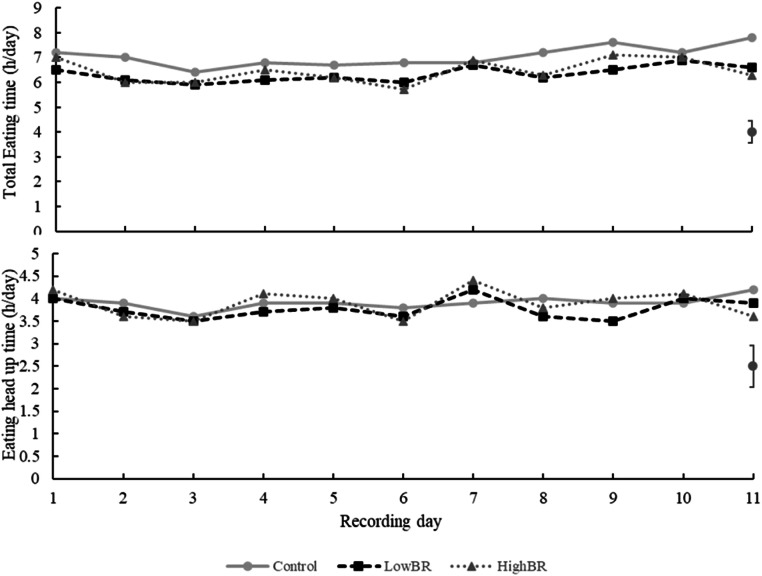
Daily total eating time and eating head up time (h/day) of dairy cows between days 18 to 28 of the experiment. CON: basal diet; LowBR: basal diet plus 106 mL/day of low-bromoform IVP; HighBR: basal diet plus 106 mL/day of high-bromoform IVP. The error bars represent the SED.

**Fig. 2:**
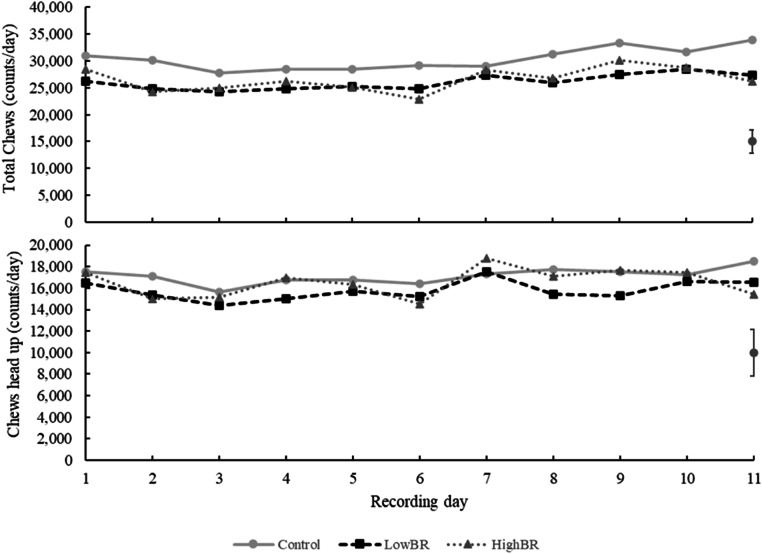
Daily total chews and chews head up time (counts/day) of dairy cows between days 18 to 28 of the experiment. CON: basal diet; LowBR: basal diet plus 106 mL/day of low-bromoform IVP; HighBR: basal diet plus 106 mL/day of high-bromoform IVP. The error bars represent the SED.

Dry matter intake was not affected by the bromoform-based IVP (*P* = 0.101), nor was eating behaviour (*P* > 0.101) as evidenced by data presented in Table 2. The duration of drinking, and other activities did not differ between cows in the CON group and cows in the bromoform-based IVP groups (LowBR and HighBR, *P* > 0.125). A treatment × day interaction was detected for the time cows spent eating with their heads up (*P* = 0.039), total eating time (*P* = 0.004), the number of chews with heads up (*P* = 0.056) and total chews (*P* = 0.04). However, no consistent trends were evident across the 11-day period for any of the variables (Fig. 1, Fig. 2). The bromoform rate in the IVP did not affect the time cows spent ruminating (*P* = 0.763) or rumination chews (*P* = 0.678).

**Table 2:** Eating behaviour of lactating dairy cows fed a bromoform-based Investigational Veterinary Product (IVP) at two rates (LowBR and HighBR), between days 18 and 28 of the experiment.

Parameter	Treatment 1				Contrast P-values			
	CON	LowBR	HighBR	SEd	CON vs BR^2^	LowBR vs HighBR	Day	Treat x Day
Number of cows	9	8	7					
Total intake (kg DM)	23.2	23.7	21.7	0.88	0.101	0.771	<0.001	0.282
Eating head up time (h/day)	3.9	3.8	3.9	0.46	0.850	0.798	<0.001	0.039
Eating head down time (h/day)	3.1	2.6	2.6	0.43	0.131	0.979	<0.001	0.229
Eating time total (h/day)	7.0	6.3	6.5	0.44	0.101	0.771	<0.001	0.004
Drinking time (h/day)	0.10	0.10	0.10	0.04	0.792	0.731	0.068	0.443
Other activities time (h/day)	7.8	8.7	8.7	0.65	0.125	0.994	<0.001	0.290
Chews head up (counts/day)	17,145	15,776	16,524	2,172.1	0.597	0.738	<0.001	0.056
Chews head down (counts/day)	13,265	10,315	10,040	2,054.0	0.095	0.896	<0.001	0.268
Total eating Chews (counts/day)	30,410	26,092	26,565	2,184.6	0.042	0.833	<0.001	0.004
Rumination chews (counts/day)	36,327	33,811	34,786	2,279.5	0.311	0.678	<0.001	0.149
Rumination time (h/day)	9.1	8.9	8.8	0.34	0.387	0.763	<0.001	0.166

1 CON: basal diet; LowBR: basal diet plus 106 mL/day of low-bromoform IVP; HighBR: basal diet plus 106 mL/day of high-bromoform IVP.

2 BR –diets containing bromoform.

### 3.2 Eating indices

Adding the bromoform-based IVP to the diet had no effect on the calculated indices for eating (*P* = 0.232), rumination (*P* = 0.774) or chewing (*P* = 0.342, Table 3). There were also no differences in the number of chews per unit of DMI (*P* = 0.177).

**Table 3:** Chewing indices of lactating dairy cows fed a bromoform-based Investigational Veterinary Product (IVP) at two rates (LowBR and HighBR), between days 18 and 28 of the experiment.

Parameter	Treatment 1				Contrast P-values			
	CON	LowBR	HighBR	SEd	CON vs BR^2^	LowBR vs HighBR	Day	Treat x Day
Number of cows	9	8	7					
Eating Index (h/kg DMI)	0.31	0.27	0.30	0.021	0.232	0.159	<0.001	0.377
Ruminating Index (h/kg DMI)	0.40	0.38	0.41	0.021	0.774	0.090	<0.001	0.295
Chewing Index (h/kg DMI)	0.70	0.70	0.70	0.030	0.342	0.084	<0.001	0.538
Chews per unit DMI (counts/kg DM)	2939	2558	2872	186.2	0.177	0.114	<0.001	0.598

1 CON: basal diet; LowBR: basal diet plus 106 mL/day of low-bromoform IVP; HighBR: basal diet plus 106 mL/day of high-bromoform IVP.

2 BR –diets containing bromoform.

### 3.3 Locomotion behaviour

The presence of the bromoform-based IVP in the diet did not affect the number of hours per day cows spent lying (*P* = 0.853) or standing (*P* = 0.679). Cows on the CON diet spent less time walking per day compared to those in the HighBR treatment group (*P* = 0.010, Table 4).

**Table 4:** Locomotion behaviour of lactating dairy cows fed a bromoform-based Investigational Veterinary Product (IVP) at two rates (LowBR and HighBR), between days 18 and 28 of the experiment.

Parameter	Treatment 1				Contrast P-values			
	CON	LowBR	HighBR	SEd	CON vs BR^2^	LowBR vs HighBR	Day	Treat x Day
Number of cows	9	9	7					
Lying time (h/day)	9.6	9.8	9.6	0.79	0.853	0.775	<0.001	0.806
Standing time (h/day)	13.3	12.9	13.1	0.78	0.679	0.814	<0.001	0.818
Walking time (h/day)	1.3^a^	1.4^ab^	1.5^b^	0.07	0.010	0.518	<0.001	0.211

1 CON: basal diet; LowBR: basal diet plus 106 mL/day of low-bromoform IVP; HighBR: basal diet plus 106 mL/day of high-bromoform IVP.

2 BR –diets containing bromoform.

a,b Means within a row followed by different superscripts are significantly different (*P* < 0.05).

### 3.4 Circadian patterns of eating, rumination, and other activity

**Fig. 3:**
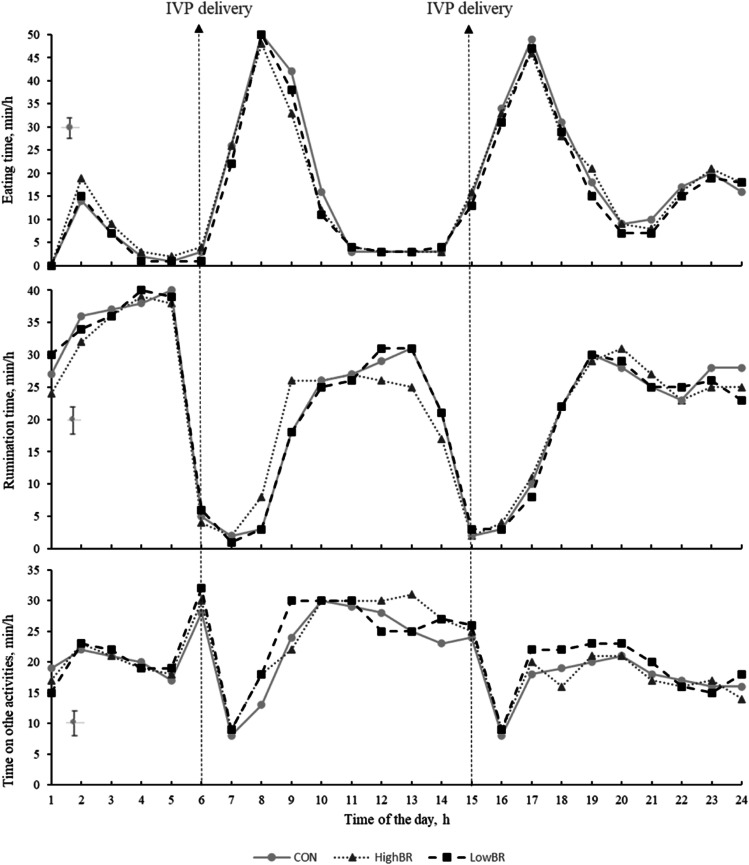
Daily fluctuation in eating, rumination and other activity behaviours of dairy cows between days 18 to 28 of the experiment. CON: basal diet; LowBR: basal diet plus 106 mL/day of low-bromoform IVP; HighBR: basal diet plus 106 mL/day of high-bromoform IVP. The dotted arrows represent the grain feeding time during milking at 6 AM and 3 PM. The error bars represent the SED.

There were no differences in the circadian patterns of eating (*P* = 0.251), rumination (*P* = 0.291) or other activities (*P* = 0.179) (Fig. 3) between cows on the CON treatment and cows on the bromoform-based IVP treatments.

## 4 Discussion

### 4.1 Eating and rumination

Feeding cows an IVP with two different concentrations of a synthetic bromoform additive had no impact on their eating and rumination behaviour. Thus, we accept our first hypothesis. Our results are in contrast to those of Nyløy et al. (2023), who observed that lactating dairy cows fed *A. taxiformis* at 0.25 % organic matter inclusion in a total mixed ration (TMR) diet had greater eating time, greater chewing index and a 10 % reduction in DMI compared to the control group. This difference may be attributed to other components present in seaweed-based products, as opposed to synthetic bromoform. To our knowledge, no other prior studies have examined the effects of halogenated compounds on animal behaviour. In contrast, a small number of studies have investigated the effects of 3-NOP, a compound that is also a direct inhibitor of the methanogenesis pathway, on animal feeding behaviour (Vyas et al., 2018; Kim et al., 2019; Maigaard et al., 2024). Maigaard et al. (2024) observed altered feeding behaviour when 76 mg/kg DM of 3-NOP was fed to dairy cows in a partial mixed ration, exhibiting a greater frequency of smaller meals, slower eating rates, and a 13.4 % reduction in DMI. They also found that the number of attempts to visit unassigned feed bins (those not designated for the cow) and the percentage of those visits relative to total daily visits increased additively when fat, nitrate, and 3-NOP were included in the diet. Consistent with our findings, Vyas et al. (2018) and Kim et al. (2019) observed no effects on animal behaviour when 3-NOP was fed to beef steers. Vyas et al. (2018) reported no changes in meal duration, eating duration, or meal frequency when beef steers were supplemented with 3-NOP at 200 mg/kg DM during the backgrounding period (high-forage diet) or at 125 mg/kg DM during the finishing phase (high-grain diet). Similarly, dietary supplementation of 100 mg/kg DM of 3-NOP did not affect feeding behaviour in beef steers on either a high-forage or a high-grain diet (Kim et al., 2019). While our study and that of Kim et al. (2019) found no differences in feeding behaviour or DMI, Vyas et al. (2018) reported that, although there were no differences in feeding behaviour, DMI decreased when 3-NOP was added to the diet.

Several factors may influence discrepancies in eating behaviour responses across studies. While the behavioural monitoring systems used (GrowSafe system (Vyas et al., 2018), Insentec RIC system (Maigaard et al., 2024), video recording (Kim et al., 2019) or RumiWatch system (Nyløy et al., 2023; present study) differ in resolution and the type of parameters captured, these methodology differences alone are unlikely to explain these contrasting outcomes. Differences in cattle species, feeding management, diet quality, and type of feed additive are also factors that might have contributed to the variability in behavioural responses among studies.

Changes in the palatability of the diet due to the addition of halogenated compounds (Roque et al., 2019) or 3-NOP (Lee et al., 2019) have been suggested as a possible factor affecting eating behaviour and, consequently, DMI. Maigaard et al. (2024) associated increased visits to unassigned bins and negative body weight changes with possible 3-NOP palatability issues, while Nyløy et al. (2023) speculated that 400 g of molasses was insufficient to mask the taste of *A.taxiformis*. In the study by Nyløy et al. (2023), cows probably used the taste as a cue to sort out the *A. taxiformis*, spending more time eating head down while selecting against *A. taxiformis.* In our study, no grain refusals were observed, indicating that 227 and 455 mg bromoform per head per day did not affect the palatability of the grain component of the diet to the point that animals refused offered grain.

Beyond palatability, physiological discomfort may also alter eating behaviour. The use of methane mitigants has been associated with increased hydrogen production (Hristov et al., 2015; Vyas et al., 2018; Roque et al., 2019), elevated lactate, formate and ethanol concentrations (Melgar et al., 2020a), and raised ruminal hydrogen partial pressure (Martinez-Fernandez et al., 2017), which have been linked to reductions in DMI (Ungerfeld, 2018) and potential rumen discomfort or malaise, further raising animal welfare concerns.

An interaction effect between treatment and day was detected for eating and chewing behaviour variables. However, when examining the plotted data across days and treatments, no clear trend, adaptation to the product, or abnormal pattern was evident. The absence of a consistent trend suggests that the interaction may reflect day-to-day variation rather than a biological response to the treatments.

The eating times recorded in our study are close to those reported in previous studies for dairy cows eating lucerne hay with comparable neutral detergent fibre concentration (364 min/day, Beauchemin et al., 1994). However, a recent meta-analysis (White et al., 2017) did report highly variable eating (ranging from 141 to 507 min/day) and ruminating (ranging from 236 to 610 min/day) time results, with our findings falling within these reported ranges. This variability might be attributed to the measurement employed, DMI, the physical and chemical composition of the diet (Beauchemin, 2018), and individual animal variability (De Boever et al., 1990). Further studies are required to understand the impact of halogenated compounds, including bromoform, in different management systems (e.g. TMR vs. grazing) and on different diet compositions.

A decline in rumination time has been established as a reliable predictor of health issues (Herskin et al., 2004), cow stress and metabolic disorders (Stangaferro et al., 2016), with significant implications for animal welfare. In our study, the bromoform-based IVP did not affect rumination time, indicating that its inclusion in the diet did not compromise cow well-being under the conditions implemented in this study.

Drinking behaviour was not affected in our study, aligning with the lack of observed changes in eating and rumination behaviour. It could have been hypothesised that drinking behaviour would have been affected to overcome discomfort in those studies where eating behaviour was affected. However, neither Maigaard et al. (2024) nor Nyløy et al. (2023) found changes in water intake or drinking behaviour associated with supplemented 3-NOP or seaweed containing bromoform. It is important to highlight these results as water intake plays a key role in animal health, performance and welfare (Murphy, 1992; Meyer et al., 2004).

Chewing activity stimulates salivary secretion and reduction of feed particle size, increasing nutrient degradation and preserving rumen health (Allen, 1997), while also maintaining high levels of DMI and efficient digestive function (Beauchemin, 2018). In our experiment, cows fed the bromoform-based IVP showed fewer total chews per day than control cows, but values remained within the expected range for dairy cows (12,000 and 30,000 times during eating and 20,000 to 40,000 times during rumination), depending on diet characteristics and chewing duration (Beauchemin & Buchanan-Smith, 1989; Dado & Allen, 1994). In contrast, Nyløy et al. (2023) reported increased chewing activity from cows supplemented with *A. taxiformis* and attributed these changes to cows sorting and selecting against *A. taxiformis* due to its taste, as it adhered to the concentrate portion of the TMR, resulting in a higher DMI of the silage component of the diet. The astringency of *A. taxiformis* may have also contributed to the increased chewing and saliva secretion, reflecting effects of the additive itself rather than the active compound bromoform. Further research is required to understand the effect of the bromoform-based IVP on chewing behaviour.

Eating, ruminating, and chewing indices did not differ when the cows were fed the bromoform-based IVP at either dose. Reported chewing indices vary widely: Jensen et al. (2016) reported a range from 24.6 to 62.5 when feeding a total mixed ration, and from 30.2 to 49.4 when feeding separate ingredients. Similarly, Zebeli et al. (2006) reported a range of 17.9 to 47.1 in a meta-analysis, considering a chewing index of 30 min/kg of DM as the desirable target for optimal rumen function. Our results fall within these reported ranges, suggesting that the bromoform-based IVP did not negatively impact the cows' chewing efficiency or disrupt normal rumination processes.

### 4.2 Lying, standing and walking behaviour

We observed no effect of feeding the two concentrations of bromoform-based IVP on lying or standing time, but cows fed the CON diet spent 12 min less time walking compared with cows in the bromoform-based IVP diets. Therefore, we partially accept our second hypothesis as it is not valid for all locomotion parameters. Consistent with these findings, Kim et al. (2019) found no difference in lying or standing behaviour when 3-NOP was fed to beef cattle. Research on the effects of methane inhibitor additives on the locomotion behaviour of dairy cattle is limited, highlighting the need for further studies in this area.

The lying durations in our study were comparable to those reported for dairy cows under grazing conditions (8.5 h/day, Sepúlveda-Varas et al., 2014; 9.5 h/day, Beggs et al., 2018), but slightly below the recommended thresholds set as 10 to 12 h/day by the Ministry for Primary Industries New Zealand (2019) or 12 h/day by the National Farm Animal Care Council Canada (2009). However, these thresholds should be viewed as guidelines rather than strict welfare indicators. Shorter lying times do not necessarily indicate compromised animal welfare, nor do longer lying times automatically ensure good welfare.

We speculate that this discrepancy may be due to the experimental conditions, where cows only had access to a bare loafing area, which may not have been ideal for optimising lying time and may not reflect how cows rest during the day in a pasture-based grazing setting. Moreover, the spatial separation of feed and water and competition among cows for preferred feed bins may have increased walking time and reduced lying opportunities. As highlighted by Tucker et al. (2021), lying behaviour is a high priority for cattle, and restrictions, whether caused by extended standing periods (Munksgaard & Simonsen, 1996; Norring & Valros, 2016) or unfavourable conditions (Fisher et al., 2003; Schütz & Cox, 2014; Schütz et al., 2019), pose significant risks to their welfare, including physiological stress responses. These findings emphasise the need to ensure adequate lying time for dairy cows, and it is notable that the bromoform-based IVP used in our study did not alter this critical behaviour.

While walking durations were statistically different between cows fed the control diet and those in the HighBR group, the practical implication of this difference is negligible, as it amounts to only 12 min/day, approximately 13 % of the cows’ total daily walking time. Given the small magnitude of this difference, it is unlikely to have any meaningful impact on the cows' overall behaviour or welfare.

### 4.3 Circadian patterns

The circadian patterns of eating, rumination, and time spent on other activities showed no differences between control cows and cows fed the bromoform-based IVP. There were two main eating sessions during the day: one after the morning milking and another after the afternoon milking, with a smaller eating session occurring overnight. This pattern may have been influenced by the cows receiving the grain at the dairy during milking, after which they walked to the automatic feeders, spending considerable time eating there, before moving to the loafing areas. Consistent with previous studies (Grant et al., 1990; Paudyal et al., 2016), most rumination occurred at night when cows were resting or during periods when they were not interrupted by management activities throughout the day. Rumination time has been reported to peak approximately four hours after eating (Schirmann et al., 2012), which aligns with our results. The observed eating and rumination trends are consistent with previous grazing studies (Bovolenta et al., 2005; Sheahan et al., 2011; Romanzin et al., 2018), suggesting that the timing of our cows’ activities is comparable with those in a grazing system. These findings provide confidence that the data generated with our feeding system effectively replicates eating patterns characteristic of a grazing system.

## 5 Conclusion

Feeding an IVP containing two rates of bromoform to lactating dairy cows twice daily at milking had no impact on their eating and rumination behaviour. While there was no effect of feeding the bromoform-based IVP on lying or standing time, cows fed the CON diet spent less time walking compared with cows in the bromoform-based IVP diets. However, the practical implication of a marginal increase of 12 min/day in walking time among IVP-fed cows is negligible. Moreover, the circadian patterns of eating, rumination, and time spent on other activities showed no differences between control cows and cows fed the bromoform-based IVP. Thus, we conclude that feeding the IVP at milking, containing up to 455 mg of bromoform per day, does not affect animal behaviour, nor cause any stress or discomfort. Also, these results suggest that the overall health and well-being of the animals remains unaffected, as our animal behaviour measurements are reliable indicators of these factors.

## Ethics statement

Cows were cared for according to the Australian Code of Practice for the Care and Use of Animals for Scientific Purposes (NHMRC, 2013). Animal use was approved by the DEECA Agricultural Research and Extension Animal Ethics Committee (approval 2022-14, 13 December 2022).

## Financial support statement

This research was funded by Agriculture Victoria and Rumin8 Pty Ltd.

## CRediT authorship contribution statement

**R. Tognelli:** Writing – review & editing, Writing – original draft, Methodology, Investigation, Data curation. **P.S. Alvarez-Hess:** Writing – review & editing, Supervision, Resources, Project administration, Methodology, Investigation, Conceptualization. **A.S. ó Neachtain:** Writing – review & editing, Investigation, Data curation. **S. Chandra:** Visualization, Methodology, Formal analysis. **S.R.O. Williams:** Writing – review & editing, Visualization, Methodology, Investigation, Data curation, Conceptualization. **S. Jacques:** Writing – review & editing. **S.E. Denman:** Writing – review & editing, Supervision. **R.J. Eckard:** Writing – review & editing, Supervision. **J.L. Jacobs:** Writing – review & editing, Supervision, Resources, Project administration, Methodology, Funding acquisition, Conceptualization.

## Declaration of competing interest

The authors declare the following financial interests/personal relationships which may be considered as potential competing interests: J.L. Jacobs reports financial support and equipment, drugs, or supplies were provided by Rumin8 Pty Ltd. Silke Jacques reports a relationship with Rumin8 Pty Ltd that includes: employment. If there are other authors, they declare that they have no known competing financial interests or personal relationships that could have appeared to influence the work reported in this paper.

## Data Availability

None of the data are deposited in an official repository. All data are available on reasonable request to the corresponding author.
